# Second primary cancer risk - the impact of applying different definitions of multiple primaries: results from a retrospective population-based cancer registry study

**DOI:** 10.1186/1471-2407-14-272

**Published:** 2014-04-18

**Authors:** Aishah Coyte, David S Morrison, Philip McLoone

**Affiliations:** 1 Undergraduate Medical School, School of Medicine, University of Glasgow, Glasgow, UK; 2 West of Scotland Cancer Surveillance Unit, Public Health Research Group, Institute of Health and Wellbeing, University of Glasgow, Glasgow, UK

**Keywords:** Second primary cancer, Relative risk, Survival, Scotland, Cancer registry

## Abstract

**Background:**

There is evidence that cancer survivors are at increased risk of second primary cancers. Changes in the prevalence of risk factors and diagnostic techniques may have affected more recent risks.

**Methods:**

We examined the incidence of second primary cancer among adults in the West of Scotland, UK, diagnosed with cancer between 2000 and 2004 (n = 57,393). We used National Cancer Institute Surveillance Epidemiology and End Results and International Agency for Research on Cancer definitions of multiple primary cancers and estimated indirectly standardised incidence ratios (SIR) with 95% confidence intervals (CI).

**Results:**

There was a high incidence of cancer during the first 60 days following diagnosis (SIR = 2.36, 95% CI = 2.12 to 2.63). When this period was excluded the risk was not raised, but it was high for some patient groups; in particular women aged <50 years with breast cancer (SIR = 2.13, 95% CI = 1.58 to 2.78), patients with bladder (SIR = 1.41, 95% CI = 1.19 to 1.67) and head & neck (SIR = 1.93, 95% CI = 1.67 to 2.21) cancer. Head & neck cancer patients had increased risks of lung cancer (SIR = 3.75, 95% CI = 3.01 to 4.62), oesophageal (SIR = 4.62, 95% CI = 2.73 to 7.29) and other head & neck tumours (SIR = 6.10, 95% CI = 4.17 to 8.61). Patients with bladder cancer had raised risks of lung (SIR = 2.18, 95% CI = 1.62 to 2.88) and prostate (SIR = 2.41, 95% CI = 1.72 to 3.30) cancer.

**Conclusions:**

Relative risks of second primary cancers may be smaller than previously reported. Premenopausal women with breast cancer and patients with malignant melanomas, bladder and head & neck cancers may benefit from increased surveillance and advice to avoid known risk factors.

## Background

The prevalence of patients living after a diagnosis of cancer has increased due to rising incidence and improving survival [1-3]. Patients often seek information on preventing and detecting further cancer occurrence [4,5]. There is therefore an increasing need to determine the risk of subsequent cancer and to provide appropriate surveillance and behaviour modification advice.

The risk of further primary cancers might be expected to be raised because of persisting effects of genetic and behavioural risk factors, long term side-effects of chemo- and radiotherapy, and increased diagnostic sensitivity. There is some evidence that this is the case [6]. For female breast cancer, the risk of contralateral breast cancer is 3% after 5 years [7] a four-fold increase [8,9]. Risks of second primary colorectal cancers are doubled [10] but might only be increased in tumours of the proximal colon [11]. A five-fold increase in risk of primary lung cancers following Hodgkin’s lymphoma has been reported [12] and risks of second primary head & neck cancers are raised [13].

A new evaluation of second primary cancer risk is needed for several reasons. There have been significant temporal changes in the prevalence of risk factors – such as smoking [14], alcohol consumption [15] and obesity [16] - which may affect cancer incidence among survivors of cancer. Diagnostic sensitivity has increased due to screening programmes [17,18] and the increased use of medical imaging technologies [19,20]. Registries use various rules to distinguish between cancers that are new cases and those that are an extension of an existing cancer. The criteria for defining second primary cancers have changed over time and differ between studies. Two sets of rules are widely used; the rules of the Surveillance Epidemiology and End Results (SEER) Program [21] are used mainly by North American cancer registries; the rules developed by the International Association of Cancer Registries (IACR) and the International Agency for Research on Cancer (IARC) [22,23] are used internationally, mainly for reporting. SEER takes account of histology, site, laterality and time since initial diagnosis to identify multiple primary cancers. The IARC/IACR rules are more exclusive; only one tumour is registered for an organ, irrespective of time, unless there are histological differences.

Our aim was to describe second primary cancers in a large geographically defined population over a period when increasing detection, greater diagnostic sensitivity and improved survival may have altered previous estimates of risk. We used Scottish cancer registry data, which have a high case ascertainment rate for most tumour types [24-26], and applied comprehensive and restricted definitions of second primary cancers.

## Methods

### Population

Using the Scottish Cancer Registry we identified all patients resident in the West of Scotland (population 2.4 million), aged ≥15 years who had a first diagnosis of a malignant primary cancer between January 2000 and December 2004 (n = 58,364). Diagnoses were coded to the International Classification of Diseases 10th revision (ICD-10). We ignored registrations of non-melanoma skin cancers (ICD-10 C44). A cancer was deemed to be a first incident, or index, cancer if there was no prior record of cancer since 1980 and the cancer was recorded as a malignant primary cancer (International Classification of Diseases for Oncology behaviour code 3). We excluded patients whose index cancer was diagnosed at date of death (n = 965). Six patients were excluded because date of death was recorded as preceding date of incidence. The final sample comprised 57,393 patients. We excluded the first sixty days of follow up from the main analysis because it is difficult to distinguish between synchronous and metachronous tumours during this period.

When cancer occurred at the same index cancer site we recorded the subsequent cancer as a primary cancer according to International Agency for Research on Cancer/International Association of Cancer Registries (IACR/IARC) rules [22,23] and also Surveillance Epidemiology and End Results (SEER) rules for reporting multiple primaries. We applied IARC/IACR rules using IARCcrgTools [27]. IARC/IACR only allow one tumour (depending on histologic group) per organ or pair of organs per person per lifetime. SEER rules were applied using the multiple primary and histology coding manual [28]. SEER rules take account of histology, site, laterality and time since diagnosis.

### Statistical methods

The relative risk of a second primary cancer was estimated by indirect standardisation. The person-years at risk among patients diagnosed with a first primary cancer were calculated from diagnosis until 5 years later, date of death or date of diagnosis of a second primary cancer, whichever came first. Data were stratified by site of first primary cancer, site of second primary cancer, sex and age at first diagnosis. The expected number of second cancers in each stratum was estimated by multiplying the total number of person-years by the age, sex and cancer specific incidence rate in the population of the West of Scotland in each year between 2000 and 2009. Standardised incidence ratios (SIR) were obtained by dividing the observed number of cases of second primary cancer by the number expected. This provided an estimate of the risk of a cancer patient developing a second primary cancer relative to the incidence of cancer in the West of Scotland general population. Relative risks are presented with exact 95% confidence intervals for Poisson counts. Rates of second cancer incidence were expressed per 100 person-years and age and sex standardised to the European standard population. STATA version 11 (StataCorp, CollegeStation, TX, USA) was used to conduct statistical analyses.

### Ethics

Formal ethical approval was unnecessary because the analysis employed routinely collected non-patient identifiable data. The use of these data for research purposes has been approved by the Privacy Advisory Committee to the Board of NHS National Services Scotland.

## Results

**Figure 1:**
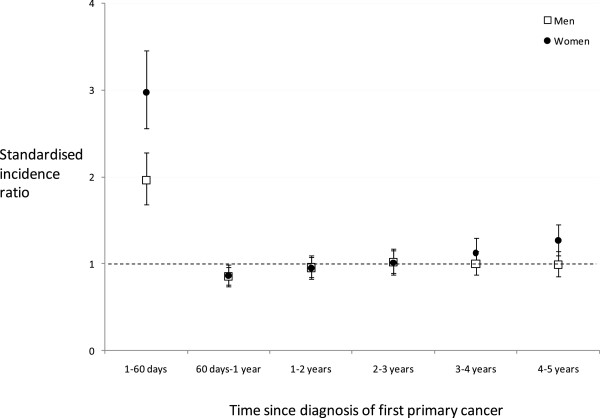
Standardised incidence ratios (SIR) for subsequent primary cancers.

We identified 57,393 patients with an incident primary cancer. Five percent (2966/57393) were diagnosed with a further primary cancer within 5 years of diagnosis. Sixteen percent of second cancers (487/2966) were diagnosed on the same day as the index cancer. A further 12% (342/2966) were diagnosed between one to sixty days after first diagnosis. The crude rate was 4.0 per 100 person-years in the first sixty days (Table 1). Over the 5 years of follow-up the rate was 2.0 per 100 person-years. Figure 1 shows the rates expressed as standardised incidence ratios (SIR) for men and women. The risk in the first 60 days was 3 times the population risk for women and 2 times the risk for men. Between 60 days and 1 year risks were lower than the reference population. The overall SIR between 1 day-5 years was 1.07 (95%CI = 1.03 to 1.11) for both sexes combined.

**Table 1:** Number of second primary cancers and person-years of follow up

**Time since primary cancer diagnosis**	Second cancers* (n)	**Person-years (years)**	**Crude rate (per 100 person-years)**	**Standarised rate† (per 100 person-years)**
0 days	487	0	-	-
1- 60 days	342	8605	4.0	2.1
61 days - 1 year	455	33365	1.4	0.7
>1 -5 years	1682	103464	1.6	0.8
0-5 years	2966	145435	2.0	1.0

*excluding non-melanoma skin cancer.

†age & sex standardised (European standard population ages 15 to 85+).

The age, sex and 5-year survival of the 56,564 patients who did not have a cancer in the first 60 days is summarised in Table 2. Forty seven percent were aged 70 and over, sixty one percent died within five years, and 2137 (3.8%) had a second primary cancer.

**Table 2:** Baseline characteristics and outcomes

	**Site of first primary cancer**																					
	All cancer* (C00-C97)		**Lung (C33-34)**		**Colorectal (C18-20)**		**Female breast (C50)**		**Prostate (C61)**		**Head & neck (C00-14, C30-32)**		**Stomach (C16)**		**Bladder (C67)**		**Oesophagus (C15)**		**Melanoma (C43)**		**Ovarian (C56)**	
	**n**	**(%)**	**n**	**(%)**	**n**	**(%)**	**n**	**(%)**	**n**	**(%)**	**n**	**(%)**	**n**	**(%)**	**n**	**(%)**	**n**	**(%)**	**n**	**(%)**	**n**	**(%)**
Total	56564		10764	(19.0)	7225	(12.8)	8386	(14.8)	5269	(9.3)	2398	(4.2)	2079	(3.7)	1832	(3.2)	1780	(3.1)	1696	(3.0)	1325	(2.3)
Men	27561	(48.7)	5894	(54.8)	3834	(53.1)			5269	(100.0)	1697	(70.8)	1262	(60.7)	1233	(67.3)	1094	(61.5)	736	(43.4)		
Mean age (SD) years	66.6	(13.8)	70.0	(10.2)	69.9	(11.7)	62.0	(14.3)	71.6	(9.1)	63.3	(12.2)	70.6	(11.7)	71.8	(10.5)	69.8	(11.6)	55.5	(18.0)	63.8	(14.7)
Age (years)																						
15– < 50	6386	(11.3)	345	(3.2)	388	(5.4)	1660	(19.8)	42	(0.8)	277	(11.6)	113	(5.4)	46	(2.5)	85	(4.8)	646	(38.1)	219	(16.5)
50– < 70	23787	(42.1)	4513	(41.9)	2868	(39.7)	4089	(48.8)	2098	(39.8)	1385	(57.8)	763	(36.7)	658	(35.9)	735	(41.3)	619	(36.5)	599	(45.2)
70+	26391	(46.7)	5906	(54.9)	3969	(54.9)	2637	(31.4)	3129	(59.4)	736	(30.7)	1203	(57.9)	1128	(61.6)	960	(53.9)	431	(25.4)	507	(38.3)
Number of patients with second primary cancer*	2137	(3.8)	119	(1.1)	324	(4.5)	363	(4.3)	342	(6.5)	204	(8.5)	46	(2.2)	140	(7.6)	26	(1.5)	102	(6.0)	20	(1.5)
Second or later cancer																						
Lung (C33-34)	507	(23.7)	24	(20.2)	65	(20.1)	67	(18.5)	91	(26.6)	88	(43.1)	13	(28.3)	50	(35.7)	6	(23.1)	14	(13.7)	3	(15.0)
Colorectal (C18-20)	315	(14.7)	11	(9.2)	56	(17.3)	52	(14.3)	80	(23.4)	17	(8.3)	8	(17.4)	15	(10.7)	6	(23.1)	9	(8.8)	1	(5.0)
Female Breast (C50)	237	(11.1)	14	(11.8)	25	(7.7)	98	(27.0)		(0.0)	5	(2.5)	6	(13.0)	3	(2.1)	0	(0.0)	15	(14.7)	6	(30.0)
Prostate (C61)	162	(7.6)	11	(9.2)	45	(13.9)		(0.0)	3	(0.9)	11	(5.4)	3	(6.5)	39	(27.9)	1	(3.8)	8	(7.8)	0	(0.0)
Head & neck (C00-C14, C30-C32)	107	(5.0)	15	(12.6)	12	(3.7)	11	(3.0)	15	(4.4)	32	(15.7)	2	(4.3)	2	(1.4)	5	(19.2)	2	(2.0)	0	(0.0)
Stomach (C16)	83	(3.9)	6	(5.0)	16	(4.9)	7	(1.9)	22	(6.4)	6	(2.9)	3	(6.5)	7	(5.0)	0	(0.0)	0	(0.0)	0	(0.0)
Bladder (C67)	78	(3.6)	8	(6.7)	9	(2.8)	6	(1.7)	20	(5.8)	9	(4.4)	2	(4.3)	1	(0.7)	2	(7.7)	3	(2.9)	0	(0.0)
Oesophagus (C15)	73	(3.4)	4	(3.4)	7	(2.2)	9	(2.5)	10	(2.9)	18	(8.8)	1	(2.2)	3	(2.1)	1	(3.8)	3	(2.9)	0	(0.0)
Melanoma (C43)	84	(3.9)	2	(1.7)	8	(2.5)	17	(4.7)	14	(4.1)	0	(0.0)	1	(2.2)	2	(1.4)	0	(0.0)	28	(27.5)	2	(10.0)
Pancreas (C25)	49	(2.3)	1	(0.8)	12	(3.7)	10	(2.8)	7	(2.0)	1	(0.5)	1	(2.2)	5	(3.6)	0	(0.0)	4	(3.9)	0	(0.0)
Ovarian (C56)	34	(1.6)	0	(0.0)	3	(0.9)	19	(5.2)		(0.0)	0	(0.0)	0	(0.0)	0	(0.0)	0	(0.0)	1	(1.0)	1	(5.0)
Kidney (C64)	52	(2.4)	6	(5.0)	7	(2.2)	7	(1.9)	11	(3.2)	4	(2.0)	1	(2.2)	0	(0.0)	1	(3.8)	2	(2.0)	0	(0.0)
Corpus uteri (C54)	42	(2.0)	1	(0.8)	9	(2.8)	20	(5.5)		(0.0)	2	(1.0)	1	(2.2)	0	(0.0)	1	(3.8)	0	(0.0)	3	(15.0)
All other cancers*	314	(13.9)	16	(12.9)	50	(14.7)	40	(10.6)	69	(19.2)	11	(4.8)	4	(8.5)	13	(8.8)	3	(11.1)	13	(11.9)	4	(19.0)
Number of deaths	34648	(61.3)	10130	(94.1)	4283	(59.3)	2429	(29.0)	2169	(41.2)	1262	(52.6)	1835	(88.3)	1157	(63.2)	1629	(91.5)	373	(22.0)	879	(66.3)

*excluding non-melanoma skin cancer.

Table 2 shows there was concordance in the order of first and second primary cancers. Lung cancer comprised 19% and 24% of first and second cancers, respectively; colorectal cancer comprised 13% and 15%; and female breast cancer represented 15% and 11%. Five year survival and the proportion of patients who had a further cancer were associated. One to two percent of patients with cancers with poor survival (6-25% alive at 5 years for lung, oesophagus, ovarian, and stomach cancer) had a further primary cancer. The proportion of second cancers among patients with cancers with better survival (37-79% alive at 5 years) ranged from 4% for colorectal to 8.5% for head & neck cancers.

Compared to the distribution of index cancers sites (Table 2), there were higher proportions of head & neck cancers (13% vs. 4% of index cancers) among patients with lung cancer, higher proportions of lung cancer (43% vs. 19%) among head & neck cancer patients, and higher proportions of prostate (28% vs. 9%) and lung cancers (36% vs. 19%) among patients with bladder cancer. P < 0.0001 for each comparison.

Later cancers at the same site may be recurrences rather than true primaries. Table 3 shows the number of registered subsequent primary cancers, in the same ICD-10 category as the index cancer, classified by IARC/IACR and SEER rules. In each case SEER included a greater number of second primaries than IARC/IACR. Female breast cancer showed the greatest difference; 79 of 98 subsequent breast cancers were classified as second primaries by SEER but only 1 by IARC. For malignant melanoma, all 28 subsequent melanomas were identified as second primaries by SEER but none by IARC/IACR rules. There was a smaller difference for head & neck cancers, for which SEER and IARC/IACR included 29 and 24 of 32 subsequent head & neck cancers.

**Table 3:** Number of same site second primary cancers

	**Number of subsequent same site primary cancers‡**		
	**Registry**	**IARC/IACR rules**	**SEER rules**
**First primary cancer**	**n**	**n**	**n**
Lung (C33-34)	24	8	17
Colorectal (C18-20)	56	31	55
Female Breast (C50)	98	1	79
Prostate (C61)	3	0	0
Head & neck (C00-C14, C30-C32)	32	24	29
Stomach (C16)	3	0	1
Bladder (C67)	1	0	1
Oesophagus (C15)	1	0	0
Melanoma (C43)	28	0	28
Pancreas (C25)	1	0	0
Ovarian (C56)	1	0	1
Kidney (C64)	3	0	2
Corpus uteri (C54)	1	0	1

*Abbreviations:SEER* Surveillance Epidemiology and End Results, *IARC* International Agency for Research on Cancer, *IACR* International Association of Cancer Registries.

‡diagnosed >60 days- 5 years after first primary cancer.

Table 4 shows the relative risk of second primary cancers using all registrations, all registrations applying IARC/IACR and SEER rules, and all registrations excluding same site cancers. There was no overall difference in cancer incidence compared to the general population (SIR = 0.96, 95% CI = 0.91 to 1.00; SIR = 0.99, 95% CI = 0.94 to 1.04 excluding same site cancers). IARC/IACR rules, which excluded the largest number of subsequent cancers, suggested a lower risk (SIR = 0.86, 95% CI = 0.81 to 0.90). Patients with lung, colorectal, breast (aged ≥50 years), prostate and ovarian cancers were at lower risk of further cancers compared with the general population. IARC/IACR and SEER rules reduced the estimated risks further. Patients with cancers of the head & neck, bladder, and breast (women aged <50 years) showed statistically significant raised rates of subsequent cancers. Patients with malignant melanoma had raised risks of second cancers using all registrations and SEER rules; however, IARC/IACR rules suggested no excess risk.

**Table 4:** Standardised incidence ratios of second primary cancers at >60 days - 5 years

	**Registry**			**IARC/IACR rules**			**SEER rules**			**Registry‡**		
**First primary cancer**	**Number with second cancer**	**SIR (95% CI)**		**Number with second cancer**	**SIR (95% CI)**		**Number with second cancer**	**SIR (95% CI)**		**Number with second cancer**	**SIR‡ (95% CI)**	
Lung (C33-34)	119	0.79	(0.65, 0.94)	105	0.69	(0.57, 0.84)	112	0.74	(0.61, 0.89)	98	0.83	(0.67, 1.01)
Colorectal (C18-20)	324	0.89	(0.80, 1.00)	301	0.83	(0.74, 0.93)	323	0.89	(0.80, 1.00)	271	0.86	(0.76, 0.97)
Female breast (C50)	363	0.96	(0.86, 1.06)	272	0.71	(0.63, 0.80)	348	0.92	(0.82, 1.02)	274	0.96	(0.85, 1.08)
Age <50	52	2.13	(1.58, 2.78)	25	1.02	(0.65, 1.50)	49	2.00	(1.48, 2.64)	28	1.67	(1.11, 2.42)
Age ≥50	311	0.88	(0.78, 0.98)	247	0.69	(0.61, 0.78)	299	0.84	(0.75, 0.94)	246	0.92	(0.81, 1.04)
Prostate (C61)	342	0.71	(0.63, 0.79)	339	0.70	(0.63, 0.78)	323	0.89	(0.80, 1.00)	339	0.88	(0.79, 0.98)
Head & neck (C00-C14, C30-C32)	204	1.93	(1.67, 2.21)	196	1.85	(1.60, 2.13)	201	1.90	(1.64, 2.18)	176	1.74	(1.49, 2.02)
Stomach (C16)	46	1.05	(0.77, 1.40)	43	0.98	(0.71, 1.32)	44	1.00	(0.73, 1.35)	43	1.02	(0.74, 1.38)
Bladder (C67)	140	1.41	(1.19, 1.67)	138	1.39	(1.10, 1.55)	140	1.41	(1.19, 1.67)	139	1.46	(1.23, 1.73)
Oesophagus (C15)	26	0.80	(0.52, 1.17)	25	0.77	(0.50, 1.13)	25	0.77	(0.50, 1.13)	25	0.80	(0.51, 1.18)
Melanoma (C43)	102	1.35	(1.10, 1.64)	75	0.98	(0.77, 1.23)	102	1.35	(1.10, 1.64)	75	1.01	(0.79, 1.27)
Ovarian (C56)	19	0.61	(0.36, 0.95)	18	0.58	(0.34, 0.91)	19	0.61	(0.37, 0.95)	18	0.60	(0.36, 0.95)
All of above	1685	0.96	(0.91, 1.00)	1512	0.86	(0.81, 0.90)	1637	1.00	(0.95, 1.05)	1458	0.99	(0.94, 1.04)

‡excluding same site.

*Abbreviations:SEER* Surveillance Epidemiology and End Results, *IARC* International Agency for Research on Cancer, *IACR* International Association of Cancer Registries, *SIR* standardised incidence ratio, *CI* confidence interval.

**Figure 2:**
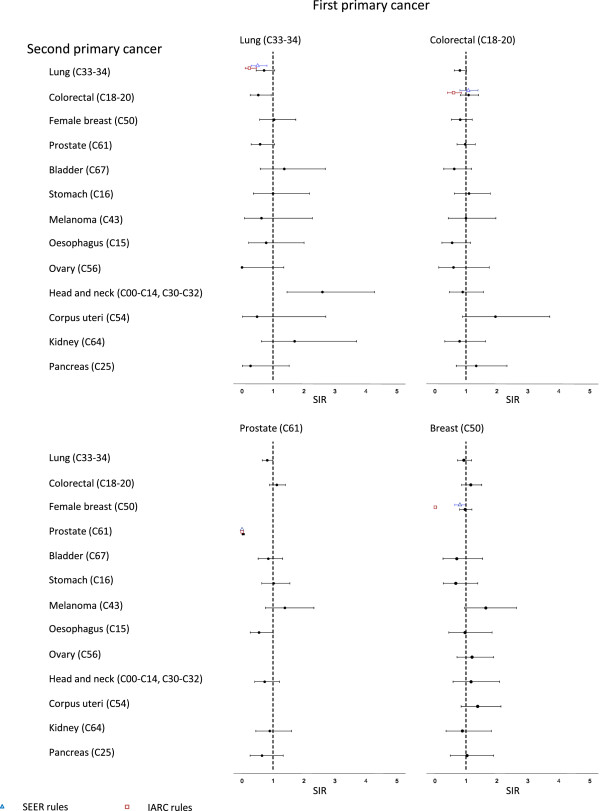
Standardised incidence ratios (SIR) for specific second primary cancers among patients with lung, colorectal, prostate and breast cancer.

**Figure 3:**
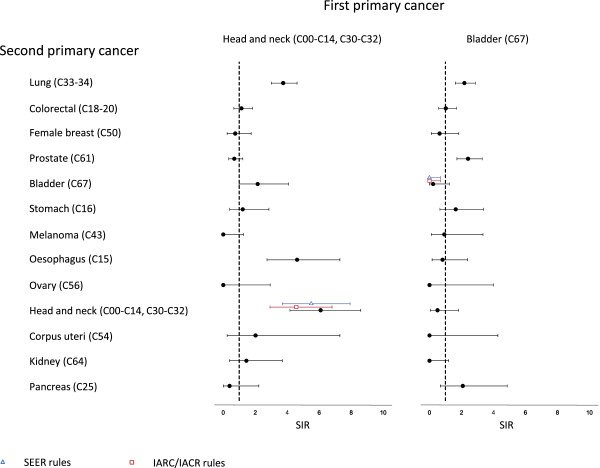
Standardised incidence ratios (SIR) for specific second primary cancers among patients with head and neck cancer, and bladder cancer.

Figures 2 and 3 shows SIRs for 13 common second or further primaries within 5 years of first diagnosis of lung, colorectal, breast, prostate, bladder and head & neck cancer. SIRs based on IARC/IACR and SEER rules are also shown. For primary lung cancer, lower risks of colorectal cancer (SIR = 0.53, 95% CI = 0.26 to 0.94) and higher risks of head & neck cancers (SIR = 2.60, 95% CI = 1.45 to 4.28) were observed (Figure 2). When IARC/IACR and SEER rules were applied, the risk of subsequent lung cancer was significantly lower. For primary colorectal cancers there were no significant differences in risk for each cancer compared to the reference population, although there was a suggestion of an increased risk of endometrial cancer (SIR = 1.95, 95% CI = 0.89 to 3.71). Applying IARC/IACR, but not SEER, rules reduced the estimated risk of subsequent colorectal cancer. Prostate cancer patients experienced lower rates of lung (SIR = 0.81, 95% CI = 0.65 to 1.00), oesophageal (SIR = 0.55, 95% CI = 0.26 to 1.01) and prostate cancers (SIR = 0.03, 95% CI = 0.01 to 0.09), and a non-significant higher rate of melanoma (SIR = 1.38, 95%CI = 0.76 to 2.32). Women with primary breast cancers also had a non-significant raised risk of malignant melanoma (SIR = 1.64, 95% CI = 0.96 to 2.63). Risks of second primary breast cancers were no different from the reference population but estimates were significantly lower (SIR = 0.01, 95% CI = 0 to 0.06) applying IARC rules. Among head & neck patients (Figure 3) there were significantly raised risks of lung cancers (SIR = 3.75, 95% CI = 3.01 to 4.62), oesophageal (SIR = 4.62, 95% CI = 2.73 to 7.29) and head & neck cancers (SIR = 6.10, 95% CI = 4.17 to 8.61). SEER or IARC/IACR rules did not significantly change SIRs for second head & neck cancers. Patients with primary bladder cancers had raised risks of cancers of the lung (SIR = 2.18, 95% CI = 1.62 to 2.88) and prostate (SIR = 2.41, 95% CI = 1.72 to 3.30).

Standardised incidence ratios for selected cancers are shown in Table 5. Between 60 days and 1 year after diagnosis, rates of second primary cancers were generally lower than the reference population. When either IARC/IACR or SEER rules were applied, rates of second primary cancers were lower than rates based on all registrations. Among patients alive one year after diagnosis, risks of further cancers were raised in women with breast cancer aged ≥50 years (SIR = 2.32, 95% CI =1.71 to 3.07) and patients with head & neck cancers (SIR =2.10, 95% CI = 1.80 to 2.44). Risks of cancers among prostate cancer survivors at 1 year were significantly lowered (SIR = 0.69, 95% CI = 0.61 to 0.78). One-to-five-year risks were similar to the general population among patients with cancers of the lung, colorectum and breast (aged ≥50 years) although IARC exclusions meant that risks were significantly lowered in patients with colorectal and breast cancers (aged ≥50 years).

**Table 5:** Standardised incidence ratios of second primary cancer by time since diagnosis

			**Registry**		**IARC/IACR**		**SEER**		**Registry‡**	
**First primary cancer**		**Time since diagnosis**	**SIR (95% CI)**		**SIR (95% CI)**		**SIR (95% CI)**		**SIR (95% CI)**	
Lung cancer (C33-34)		61 days- < 1 year	0.55	(0.39, 0.76)	0.47	(0.32, 0.66)	0.38	(0.27, 0.53)	0.58	(0.39, 0.82)
		1-5 years	0.98	(0.78, 1.22)	0.88	(0.69, 1.11)	0.95	(0.75, 1.18)	1.04	(0.81, 1.32)
Colorectal cancer (C18-20)		61 days- < 1 year	0.79	(0.61, 1.00)	0.73	(0.56, 0.94)	0.78	(0.60, 0.99)	0.81	(0.61, 1.04)
		1-5 years	0.93	(0.82, 1.05)	0.86	(0.75, 0.98)	0.93	(0.82, 1.05)	0.88	(0.77, 1.01)
Prostate cancer (C61)		61 days- < 1 year	0.77	(0.61, 0.96)	0.75	(0.59, 0.94)	0.75	(0.59, 0.94)	0.94	(0.74, 1.18)
		1-5 years	0.69	(0.61, 0.78)	0.69	(0.61, 0.78)	0.69	(0.61, 0.78)	0.87	(0.77, 0.98)
Breast cancer (C50)	*age < 50*	61 days- < 1 year	1.05	(0.29, 2.70)	0.53	(0.06, 1.90)	0.79	(0.16, 2.31)	1.11	(0.23, 3.25)
		1-5 years	2.32	(1.71, 3.07)	1.11	(0.70, 1.66)	2.22	(1.63, 2.96)	1.77	(1.15, 2.62)
Breast cancer (C50)	*age* ≥ *50*	61 days- < 1 year	0.75	(0.56, 0.98)	0.65	(0.47, 0.87)	0.68	(0.50, 0.90)	0.84	(0.61, 1.13)
		1-5 years	0.91	(0.80, 1.02)	0.70	(0.61, 0.81)	0.88	(0.77, 1.00)	1.16	(1.02, 1.32)
Head & neck (C00-C14, C30-C32)		61 days- < 1 year	1.33	(0.91, 1.88)	1.25	(0.84, 1.78)	1.29	(0.88, 1.83)	1.05	(0.67, 1.56)
		1-5 years	2.10	(1.80, 2.44)	2.02	(1.73, 2.36)	2.07	(1.77, 2.41)	1.94	(1.64, 2.27)

‡excluding same site.

*Abbreviations:SEER* Surveillance Epidemiology and End Results, *IARC* International Agency for Research on Cancer, *IACR* International Association of Cancer Registries, *SIR* standardised incidence ratio, *CI* confidence interval.

## Discussion

We found an overall raised risk of second primary cancer among patients with a first malignancy (SIR = 1.07). There was no overall increase in risk when the first sixty days were excluded. However, the risk was raised for patients with specific cancers. Women aged <50 years with cancer of the breast and patients with melanoma, bladder and head & neck cancers were at increased risk. Patients with lung cancer were at increased risk of subsequent head & neck cancers and patients with head & neck cancers were at increased risk of lung cancer, as well as oesophageal and other head & neck tumours. Patients with bladder cancers were at increased risk of lung and prostate cancer.

Studies have reported relative risks of second primary cancers ranging from 1.08 to 1.3 [6,29-31]. Patients with cancer may be at increased risk of further primary cancers for three main reasons: they are subject to intensive investigations and ongoing surveillance; genetic and behavioural risk factors for the initial cancer may persist; and treatment, particularly radiotherapy and chemotherapy, may increase the risk of future malignancies.

We found a raised risk of second cancer diagnosis during the first 60 days, suggesting an artefact of investigation. Crocetti observed a similar overall raised risk to ours (SIR = 1.08) which was lost when cancers detected in the first two months were excluded [31]. However, Youlden observed larger risks (SIRs of 1.2 and 1.4 in men and women, respectively) which remained 10 years later [30]. Curtis found that raised risks were greatest in recent years (1995–2000) which may reflect increasingly sensitive diagnostic investigation [6].

The most prevalent lung cancer risk factor is cigarette smoking. This could explain the increased risk of other smoking-related cancers among patients with lung or head & neck cancer [32]. A similar association may explain the increase in lung cancer found among patients with bladder cancer. However, clinical investigation of abnormalities in anatomically adjacent sites is likely to increase detection and this could explain the excess of prostate cancers found among patients with bladder cancer. Fabbri suggested this for increased prostate and kidney cancers among men with bladder cancer [33,34].

Mellemkjaer reported an SIR of 1.25 for all cancers in women with a primary breast cancer [35]. We found an SIR of 2.13 among women aged <50 years with breast cancer, but applying the same IARC/IACR rules as Mellemkjaier, we observed no excess risk. Women with breast cancer appeared to have a raised risk of melanoma. There have been reports of increased melanoma among patients with prostate and breast cancer [36]. A confounding factor is that prostate and breast cancers have relatively high survival and incidence is highest among socio-economic well-off groups, as is melanoma. Patients with an index cutaneous melanoma are at increased risk of second primary melanomas [37]. This is most likely due to the multifocal effects of ultraviolet light exposure on the skin.

Our finding that patients with lung, colorectal, female breast (ages ≥50 years), prostate and ovarian cancer had lower than expected numbers of later cancers is not consistent with previous reports [38-40]. This may be because we excluded cancers in the first two months, when rates have been found to be highest. Cancers detected within two months of the index cancer may otherwise have not been diagnosed until sometime later, if at all.

Our study covered a large population with a high incidence of cancer and used recent cancer registry data. Most tumour registries collect data on only malignant and in situ neoplasms. The Scottish Cancer Registry collects information on all new cases of cancer including primary malignant neoplasms, carcinoma in situ, neoplasms of uncertain behaviour and benign brain and spinal cord tumours. The guidelines for registering multiple primary cancers are not as restrictive as the IARC/IACR rules, although they have some features in common. As a general rule clinical/pathological opinions of second primary cancers are registered. IARC/IACR rules are generally used for reporting purposes. We applied both SEER and IARC/IACR rules, and explored the effect of excluding certain subsequent cancers. We did find there were cancers recorded in the registry that would be excluded as primary tumours according to IARC/IACR rules. We do not know how many additional notifications might have been included using SEER rules but were never entered onto the registry. The IARC/IACR rules may undercount multiple tumours, because cancers with the same site and histology, diagnosed more than 2 months apart but excluded as a later primary, may actually represent a new tumour. However to include these cancers could over count multiple tumours. Conversely, a new primary cancer may be misdiagnosed as a recurrence or metastasis. Our registry data found that 5% of cancer patients had a registration of a second primary cancer within 5 years. In contrast a study in the Netherlands, where the longest follow up time was 18 years, found that the percentage of patients who had a second cancer was 6% [41]. This difference might be due to differences in registration practice.

We found a similar absolute risk to others, suggesting that the smaller relative risk may be due to changing incidence in the general, comparator population. Screening programmes and initiatives to improve public awareness of cancer have contributed to greater detection; expected rates have risen and the relative risk for patients with an index cancer may have fallen. We did not have information on genetic risk factors, such as BRCA1, that might identify clusters of cancers in high risk individuals.

Patients with head & neck cancers and women <50 years old with breast cancer are at increased risk of subsequent malignancies. The pattern of second cancers was similar to that of first cancers and thus the advice to minimise exposures to the 14 lifestyle and environmental factors described by Parkin [42] remains valid. Further research is needed to determine the effects of behaviour change and previous exposure on subsequent risk. The higher rate of malignant melanoma among patients with breast and prostate cancer requires further investigation. Breast and prostate cancer are socio-economically patterned and higher rates could be due to healthcare seeking behaviour which increases detection or, in the case of melanoma, to increased sun exposure in more affluent patients. Analysis of recent second primary cancer rates in other countries is needed to test these hypotheses.

## Conclusion

The relative risk of second primary cancers may be smaller than previously reported, possibly because the general population is subject to greater surveillance and screening. Premenopausal women with breast cancer and patients with malignancies of the bladder, head & neck, and cutaneous melanoma are at increased risk of second primary cancers. It may be appropriate to offer surveillance and advice to avoid known risk factors to these patients. Further research is needed to determine whether previous perspectives of increased second primary cancer risks have been partly due to differences in detection.

## Abbreviations

IACR: International Association of Cancer Registries; IARC: International Agency for Research on Cancer; SEER: Surveillance Epidemiology and End Results; SIR: Standardised incidence ratio; CI: Confidence interval.

## Competing interests

The authors declare that they have no competing interests.

## Authors’ contributions

This paper was based on a BSc (MedSci) project by AC. The study was initially designed by DM and AC. AC carried out the original analysis. PM redesigned and reanalysed the study. All authors contributed to the interpretation of the data and the drafting of the paper. All authors read and approved the final manuscript.

## Pre-publication history

The pre-publication history for this paper can be accessed here:

http://www.biomedcentral.com/1471-2407/14/272/prepub

## References

[B1] Parkin DM Bray F Ferlay J Pisani P Global cancer statistics, 2002 CA Cancer J Clin 2005 55 74 108 10.3322/canjclin.55.2.74 15761078

[B2] Maddams J Brewster D Gavin A Steward J Elliott J Utley M Moller H Cancer prevalence in the United Kingdom: estimates for 2008 Br J Cancer 2009 101 541 547 10.1038/sj.bjc.6605148 19568236 PMC2720244

[B3] Forman D Stockton D Moller H Quinn M Babb P De Angelis R Micheli A Cancer prevalence in the UK: results from the EUROPREVAL study Ann Oncol 2003 14 648 654 http://dx.doi.org/10.1093/annonc/mdg169 10.1093/annonc/mdg169 12649115

[B4] Rutten LJ Arora NK Bakos AD Aziz N Rowland J Information needs and sources of information among cancer patients: a systematic review of research (1980–2003) Patient Educ Couns 2005 57 250 261 10.1016/j.pec.2004.06.006 15893206

[B5] Mehnert A Koch U Sundermann C Dinkel A Predictors of fear of recurrence in patients one year after cancer rehabilitation: a prospective study Acta Oncol 2013 52 1102 1109 http://dx.doi.org/10.3109/0284186X.2013.765063 10.3109/0284186X.2013.765063 23384721

[B6] Curtis RE Freedman DM Ron E Ries LAG Hacker DG Edwards BK Tucker MA Fraumeni JF Jr New malignancies among cancer survivors: SEER Cancer Registries, 1973–2000. NIH Publ. No. 05–5302 2006 Bethesda, MD: National Cancer Institute

[B7] Gao X Fisher SG Emami B Risk of second primary cancer in the contralateral breast in women treated for early-stage breast cancer: a population-based study Int J Radiat Oncol Biol Phys 2003 56 1038 1045 http://dx.doi.org/10.1016/S0360-3016(03)00203-7 10.1016/S0360-3016(03)00203-7 12829139

[B8] Soerjomataram I Louwman WJ van der Sangen MJ Roumen RM Coebergh JW Increased risk of second malignancies after in situ breast carcinoma in a population-based registry Br J Cancer 2006 95 393 397 http://dx.doi.org/10.1038/sj.bjc.6603231 10.1038/sj.bjc.6603231 16804522 PMC2360642

[B9] Chen Y Thompson W Semenciw R Mao Y Epidemiology of contralateral breast cancer Cancer Epidemiol Biomarkers Prev 1999 8 855 861 10548312

[B10] Levi F Randimbison L Blanc-Moya R Maspoli-Conconi M Rosato V Bosetti C La Vecchia C High constant incidence of second primary colorectal cancer Int J Cancer 2013 132 1679 1682 10.1002/ijc.27780 22903312

[B11] Liu L Lemmens VE De Hingh IH de Vries E Roukema JA van Leerdam ME Coebergh JW Soerjomataram I Second primary cancers in subsites of colon and rectum in patients with previous colorectal cancer Dis Colon Rectum 2013 56 158 168 10.1097/DCR.0b013e318279eb30 23303143

[B12] Ibrahim EM Kazkaz GA Abouelkhair KM Al-Mansour MM Al-Fayea TM Al-Foheidi M Bayer AM Elmasri OA Increased risk of second lung cancer in hodgkin’s lymphoma survivors: a meta-analysis Lung 2013 191 117 134 10.1007/s00408-012-9418-4 23053567

[B13] Jegu J Binder-Foucard F Borel C Velten M Trends over three decades of the risk of second primary cancer among patients with head and neck cancer Oral Oncol 2013 49 9 14 10.1016/j.oraloncology.2012.06.018 22840787

[B14] Scholes S Bajekal M Love H Hawkins N Raine R O’Flaherty M Capewell S Persistent socioeconomic inequalities in cardiovascular risk factors in England over 1994–2008: a time-trend analysis of repeated cross-sectional data BMC Public Health 2012 12 129 http://dx.doi.org/10.1186/1471-2458-12-129 10.1186/1471-2458-12-129 22333887 PMC3342910

[B15] Hotchkiss JW Davies C Gray L Bromley C Capewell S Leyland AH Trends in adult cardiovascular disease risk factors and their socio-economic patterning in the Scottish population 1995–2008: cross-sectional surveys BMJ Open 2011 1 1 e000176 http://dx.doi.org/10.1136/bmjopen-2011-000176 22021783 10.1136/bmjopen-2011-000176 PMC3191578

[B16] Howel D Waist circumference and abdominal obesity among older adults: patterns, prevalence and trends PLoS One 2012 7 e48528 http://dx.doi.org/10.1371/journal.pone.0048528 10.1371/journal.pone.0048528 23119047 PMC3485367

[B17] The NHS cancer screening programmes website [http://www.cancerscreening.nhs.uk/] Accessed: 19th March 2014

[B18] Moser K Sellars S Wheaton M Cooke J Duncan A Maxwell A Michell M Wilson M Beral V Peto R Richards M Patnick J Extending the age range for breast screening in England: pilot study to assess the feasibility and acceptability of randomization J Med Screen 2011 18 96 102 http://dx.doi.org/10.1258/jms.2011.011065 10.1258/jms.2011.011065 21852703

[B19] Groheux D Espie M Giacchetti S Hindie E Performance of FDG PET/CT in the clinical management of breast cancer Radiology 2013 266 388 405 10.1148/radiol.12110853 23220901

[B20] Chopra A Ford A De Noronha R Matthews S Incidental findings on positron emission tomography/CT scans performed in the investigation of lung cancer Br J Radiol 2012 85 e229 e237 http://dx.doi.org/10.1259/bjr/60606623 10.1259/bjr/60606623 22745208 PMC3474053

[B21] Adamo MB Johnson CH Ruhl JL Dickie LA 2012 SEER Program Coding and Staging Manual. NIH Publication number 12–5581 2012 Bethesda, MD: National Cancer Institute

[B22] IARC/ENCR/IACR Working Group International rules for multiple primary cancers Asian Pacific J Cancer Prev 2005 6 104 106 15801152

[B23] International Agency for Research on Cancer International Rules for Multiple Primary Cancers (ICD-O Third Edition). Internal Report No. 2004/02 2004 Lyon: IARC

[B24] Brewster D Crichton J Muir C How accurate are Scottish cancer registration data? Br J Cancer 1994 70 954 959 10.1038/bjc.1994.428 7947104 PMC2033548

[B25] Brewster D Muir C Crichton J Registration of lung cancer in Scotland: an assessment of data accuracy based on review of medical records Cancer Causes Control 1995 6 303 310 10.1007/BF00051405 7548717

[B26] Brewster D Muir C Crichton J Registration of colorectal cancer in Scotland: an assessment of data accuracy based on review of medical records Public Health 1995 109 285 292 10.1016/S0033-3506(95)80206-1 7667493

[B27] Ferlay J Burkhard C Whelan S Parkin DM Check and conversion programs for cancer registries (IARC/IACR Tools for Cancer Registries). IARC Technical Report No. 42 2005 Lyon: IARC Available from http://www.iacr.com.fr/TechRep42-MPrules.pdf. Accessed online 19th March 2014

[B28] Johnson CH Peace S Adamo P Fritz A Percy-Laurry A Edwards BK The 2007 Multiple Primary and Histology Coding Rules 2007 Bethesda, MD: National Cancer Institute

[B29] Dong C Hemminki K Second primary neoplasms in 633,964 cancer patients in Sweden, 1958–1996 Int J Cancer 2001 93 155 161 10.1002/ijc.1317 11410860

[B30] Youlden DR Baade PD The relative risk of second primary cancers in Queensland, Australia: a retrospective cohort study BMC Cancer 2011 11 83 http://dx.doi.org/10.1186/1471-2407-11-83 10.1186/1471-2407-11-83 21342533 PMC3052198

[B31] Crocetti E Buiatti E Falini P Multiple primary cancer incidence in Italy Eur J Cancer 2001 37 2449 2456 10.1016/S0959-8049(01)00314-8 11720842

[B32] Atienza JA Dasanu CA Incidence of second primary malignancies in patients with treated head and neck cancer: a comprehensive review of literature Curr Med Res Opin 2012 28 1899 1909 10.1185/03007995.2012.746218 23121148

[B33] Kellen E Kiyohara H Multiple primary cancer (MPC) associated with bladder cancer: an analysis of the clinical and autopsy cases in Japan Jpn J Clin Oncol 1985 15 S1 201 210 4009982

[B34] Fabbri C Ravaioli A Ravaioli A Bucchi L Balducci C Canuti D Desiderio F Foca F Panzini I Falcini F Risk of cancer of the prostate and of the kidney parenchyma following bladder cancer Tumori 2007 93 124 128 17557556 10.1177/030089160709300202

[B35] Mellemkjaer L Friis S Olsen JH Scelo G Hemminki K Tracey E Andersen A Brewster DH Pukkala E McBride ML Kliewer EV Tonita JM Kee-Seng C Pompe-Kirn V Martos C Jonasson JG Boffetta P Brennan P Risk of second cancer among women with breast cancer Int J Cancer 2006 118 2285 2292 10.1002/ijc.21651 16342146

[B36] Yang GB Barnholtz-Sloan JS Chen Y Bordeaux JS Risk and survival of cutaneous melanoma diagnosed subsequent to a previous cancer Arch Dermatol 2011 147 1395 1402 http://dx.doi.org/10.1001/archdermatol.2011.1133 10.1001/archdermatol.2011.1133 22184761

[B37] Schmid-Wendtner MH Baumert J Wendtner CM Plewig G Volkenandt M Risk of second primary malignancies in patients with cutaneous melanoma Br J Dermatol 2001 145 981 985 10.1046/j.1365-2133.2001.04507.x 11899153

[B38] Thellenberg C Malmer B Tavelin B Gronberg H Second primary cancers in men with prostate cancer: an increased risk of male breast cancer J Urol 2003 169 1345 1348 10.1097/01.ju.0000056706.88960.7c 12629357

[B39] Hemminki K Li X Dong C Second primary cancers after sporadic and familial colorectal cancer Cancer Epidemiol Biomarkers Prev 2001 10 793 798 11440965

[B40] Hemminki K Aaltonen L Li X Subsequent primary malignancies after endometrial carcinoma and ovarian carcinoma Cancer 2003 97 2432 2439 10.1002/cncr.11372 12733142

[B41] Liu L de Vries E Louwman M Aben K Janssen-Heijnen M Brink M Coebergh JW Soerjomataram I Prevalence of multiple malignancies in the Netherlands in 2007 Int J Cancer 2011 128 1659 1667 http://dx.doi.org/10.1002/ijc.25480 10.1002/ijc.25480 20503267

[B42] Parkin DM The fraction of cancer attributable to lifestyle and environmental factors in the UK in 2010 Br J Cancer 2011 105 S2 S5 http://dx.doi.org/10.1038/bjc.2011.474 22158314 10.1038/bjc.2011.474 PMC3252063

